# PIK3CA regulates development of diabetes retinopathy through the PI3K/Akt/mTOR pathway

**DOI:** 10.1371/journal.pone.0295813

**Published:** 2024-01-09

**Authors:** Ruijuan Guan, Zefeng Kang, Ling Li, Xin Yan, Tianpeng Gao

**Affiliations:** 1 Ophthalmology Department, Qinghai Provincial People’s Hospital, Xining, Qinghai Province, China; 2 Eye Hospital, China Academy of Chinese Medical Sciences, Beijing, China; Duke University School of Medicine, UNITED STATES

## Abstract

**Objective:**

To explore their association with the development of diabetes retinopathy (DR), single nucleotide polymorphism (SNP) mutations were screened out by high-throughput sequencing and validated in patients diagnosed with DR. To understand the role of PIK3CA in the pathogenesis of DR and explore the relationship between PIK3CA,phosphatidylinositol 3-kinase (PI3K)/protein kinase B (AKT)/mammalian target of rapamycin (mTOR),and DR, the effect of PIK3CA.rs17849079 mutation was investigated in a DR cell model.

**Methods:**

Twelve patients diagnosed with DR at the Qinghai Provincial People’s Hospital from September 2020 to June 2021 were randomly selected as the case group, while 12 healthy subjects of similar age and gender who underwent physical examination in Qinghai Provincial People’s Hospital physical examination center during the same period were randomly selected as the control group. Blood samples (2 mL) were collected from both groups using EDTA anticoagulant blood collection vessels and frozen at −20°C for future analysis. SNP mutations were detected by high-throughput sequencing, and the shortlisted candidates were subjected by Gene Ontology (GO) and Kyoto Encyclopedia of Genes and Genomes (KEGG) enrichment analyses. The detected SNP candidates were verified by expanding the sample size (first validation: 56 patients in the case group and 58 controls; second validation: 157 patients in the case group and 96 controls). A lentivirus vector carrying mutated or wild-type PIK3CA.rs17849079 was constructed. ARPE-19 cells were cultured in a medium supplemented with 10% fetal bovine serum (FBS) to establish a DR cell model. PIRES2-PIK3CA-MT and PIRES2-PIK3CA-WT vectors were transfected into DR model cells, which were categorized into control, mannitol, model, empty vector, PIK3CA wild-type, and PIK3CA mutant-type groups. Cell activity was detected by the cell counting kit (CCK)-8 assay, and cellular apoptosis was evaluated by flow cytometry. Glucose concentration and levels of cytokines tumor necrosis factor (TNF)-α and interleukin (IL)-1β were detected using enzyme-linked immunosorbent assay kits. The expression of *PIK3CA*, *AKT1*, *mTOR*, and *VEGF* genes was detected by real-time quantitative polymerase chain reaction (RT-qPCR), while the expression of PI3K, p-PI3K, AKT1, p-AKT1, mTOR, p-mTOR, and VEGF proteins was detected by western blotting.

**Results:**

The mutated SNPs were mainly enriched in the PI3K/AKT pathway, calcium ion pathway, and glutamatergic synaptic and cholinergic synaptic signaling pathways. Seven SNPs, including PRKCE.rs1533476, DNAH11.rs10485983, ERAP1.rs149481, KLHL1.rs1318761, APOBEC3C.rs1969643, FYN.rs11963612, and KCTD1.rs7240205, were not related to the development of DR. PIK3CA.rs17849079 was prone to C/T mutation. The risk of DR increased with the presence of the C allele and decreased in the presence of the T allele. High glucose induced the expression of *PIK3CA* and *VEGF* mRNAs as well as the expression of PI3K, p-PI3K, p-AKT1, p-mTOR, and VEGF proteins in ARPE-19 cells, which led to secretion of inflammatory factors TNF-αand IL-1, cell apoptosis, and inhibition of cell proliferation. The PIK3CA.rs17849079 C allele accelerated the progression of DR. These biological effects were inhibited when the C allele of PIK3CA.rs17849079 was mutated to T allele.

**Conclusion:**

The mutated SNP sites in patients with DR were mainly enriched in PI3K/AKT, calcium ion, and glutamatergic synaptic and cholinergic synaptic signaling pathways. The rs17849079 allele of PIK3CA is prone to C/T mutation where the C allele increases the risk of DR. High glucose activates the expression of *PIK3CA* and promotes the phosphorylation of PI3K, which leads to the phosphorylation of AKT and mTOR. These effects consequently increase VEGF expression and accelerate the development of DR. The C to T allele mutation in PIK3CA.rs17849079 can play a protective role and reduce the risk of DR.

## Introduction

Diabetic retinopathy (DR) is the most common microvascular complication of diabetes mellitus (DM),characterized by microaneurysm formation, microvascular obstruction, and neovascularization [1–3]. In DR, intracellular hyperglycemia damages the vascular endothelium via dysregulation of multiple pathophysiological processes [4]. Vascular endothelial growth factor (VEGF) is an important angiogenic molecule related to the progression of DR [5] that acts on endothelial cells through the phosphatidylinositol 3-kinase (PI3K) pathway [6]. Studies have highlighted the role of the PI3K/protein kinase B (AKT) pathway in cancer and the complex relationship between PI3K/AKT upstream genes and downstream factors and VEGF. We speculate the involvement of the PI3K/AKT pathway in the pathogenesis of DR.

In this study, peripheral blood samples were collected from patients with DR for sequencing and the SNPS associated with DR were searched. The SNPs with significant differences were identified, and then the sample size was expanded for validation. Cell experiments were performed to explore the relationship between screened SNPs and DR.

## Material and methods

### Ethical approval

Written informed consent was obtained from all donors as per the principles of the Declaration of Helsinki. The study was performed in compliance with relevant Chinese laws and institutional guidelines. All protocols were approved by the institutional ethics committee of the Qinghai Provincial People’s Hospital (approval number: 2020–104).

### Clinical information of patients

Twelve patients diagnosed with DR at the Qinghai Provincial People’s Hospital from September 2020 to June 2021 were randomly selected as the case group. In addition, 12 healthy subjects of similar age and gender who underwent physical examination at the Qinghai Provincial People’s Hospital physical examination center during the same period were randomly selected as the control group. The diagnosis was carried out as per the International diabetic retinopathy diagnostic criteria [7]. Inclusion criteria were as follows: (1) Patients who met the international diagnostic criteria for diabetic retinopathy and (2) long-term residents of areas above 2100 m. Duration of residence (≥10 years). Exclusion criteria were as follows: (1) Participants were excluded if they had acute complications of diabetes and co-infection. (2) Those with severe cardiovascular and cerebrovascular complications. (3) Patients with recent trauma or surgery. (4) Patients with long-term liver and kidney diseases and other endocrine diseases. (5) Pregnant women. (6) Patients with hypertensive retinopathy, high myopic retinopathy, and other fundus diseases.

### Sample collection and DNA extraction

Two milliliters of peripheral blood sample was collected from each participant. After anticoagulant treatment, the sample was frozen at −80°C before analysis. The DNA extraction process was carried out according to the instructions of a DNA extraction kit (DP335, Beijing Tiangen).

### SNP genotyping

The quality and quantity of the purified DNA were determined by measuring the absorbance at 260 nm/280 nm (A260/A280) using Nanodrop One (Thermo). DNA samples were sequenced by Illumina sequencing, and the resulting data were analyzed by Genome Studio.

### Selection of SNPs

The software PLINK 1.09 was used to perform quality control on the genotyping results. Samples with genotyping call rates < 95% were excluded. According to the Hardy-Weinberg genetic equilibrium testing, gene frequencies were substituted to obtain genotype equilibrium frequencies and then multiplied by the total population to obtain the estimation. χ^2^ test was conducted to compare observations and estimations, and the SNPs with Hardy-Weinberg equilibrium *P* value <1×10^−5^ and linkage disequilibrium (LD) r^2^≥0.8 with respect to the controls were removed. Then, pairwise identity by state (IBS) potential genetic kinship checks were conducted on all successfully genotyped samples. On the identification of a first- or second-degree relative pair, one of the two related individuals were removed. The remaining samples were then evaluated for population outliers and stratification using principal component analysis (PCA)-based methods.

### Validation of SNPs by quantitative polymerase chain reaction (qPCR)

To clarify the distribution difference of the differential SNP sites between the case and control groups, we expanded the DR patient cohort to 56 cases and the control cohort to 58 cases for the first validation analysis. For the second validation, we further expanded the DR patients to 157 cases and the control group to 97 cases.

The identified SNPs were subjected to real-time (RT)-qPCR to assess the validity of the DNA-sequencing data. S1 Table lists the primers specific for each SNP used in RT-qPCR conducted using total RNA samples. PCR amplification for each SNP was performed with three technical replicates. Glyceraldehyde-3-phosphate dehydrogenase (*GAPDH*) was used as a reference gene. The expression levels of all genes were normalized to that of *GAPDH* and calculated using the 2−ΔΔCT method.

### Cell experiment

#### Cell culture

A DR cell model was established by treating normally cultured ARPE-19 cells with 25 mM mannitol for 48 h.

#### Target sequence construction

Wild and mutant plasmids were constructed from PIK3CA.rs17849079 gene information (PIK3CA gene ID: 5290, mutant size: exon, size of sequence: 1022 bp, mutation site: rs17849079). The inserted fragment was 813 bp. PIRES2-PIK3CA-MT and PIRES2-PIK3CA-WT vectors were synthesized and transfected into the model cells.

#### Grouping of cells

The cells were categorized in the following groups: Control group, normally cultured ARPE-19 cells; mannitol group, normally cultured ARPE-19 cells treated with 25 mM mannitol for 48 h; DR model group, ARPE-19 cells cultured normally and treated with 25 mM glucose (high glucose) for 48 h; Empty vector group, ARPE-19 cells cultured normally, treated with 25 mM glucose (high glucose) for 48 h, and then transfected with the blank vector; PIK3CA wild-type group (PIK3CA wt group), ARPE-19 cells cultured normally, treated with 25 mM glucose (high glucose) for 48 h, and then transfected with the PIK3CA wild-type vector; PIK3CA mutant-type group (PIK3CA mut group), ARPE-19 cells cultured normally, treated with 25 mM glucose (high glucose) for 48 h, and then transfected with the PIK3CA mutant vector.

Cell activity was detected by the cell counting kit-8 (CCK-8) assay, and the rate of apoptosis was analyzed by flow cytometry. Glucose level was detected by a glucose assay kit, and the levels of cytokines tumor necrosis factor (TNF)-α and interleukin (IL)-1β were determined by enzyme-linked immunosorbent assay (ELISA) kits, according to the instructions of the kits (DP335, Beijing Tiangen).

*RT-qPCR*. The expression of *PIK3CA*, *AKT1*, *mTOR*, and *VEGF* genes was detected by RT-qPCR.

*Western blot analysis*. The protein expression of PI3K, p-PI3K, AKT1, p-AKT1, mTOR, p-mTOR, and VEGF was analyzed by western blotting. The details of the antibodies are shown in S2 Table.

### Statistical analyses

Statistical analyses were performed using the statistical software SPSS (version 20; SPSS Inc., Chicago, IL, USA). GraphPad Prism 5 was used to produce graphs. Data for continuous variables were expressed as mean ± standard deviation (SD). Student’s *t*-test or Mann-Whitney test was used, as appropriate, to compare two groups of independent samples.

## Results

### SNP microarray results

**Fig 1:**
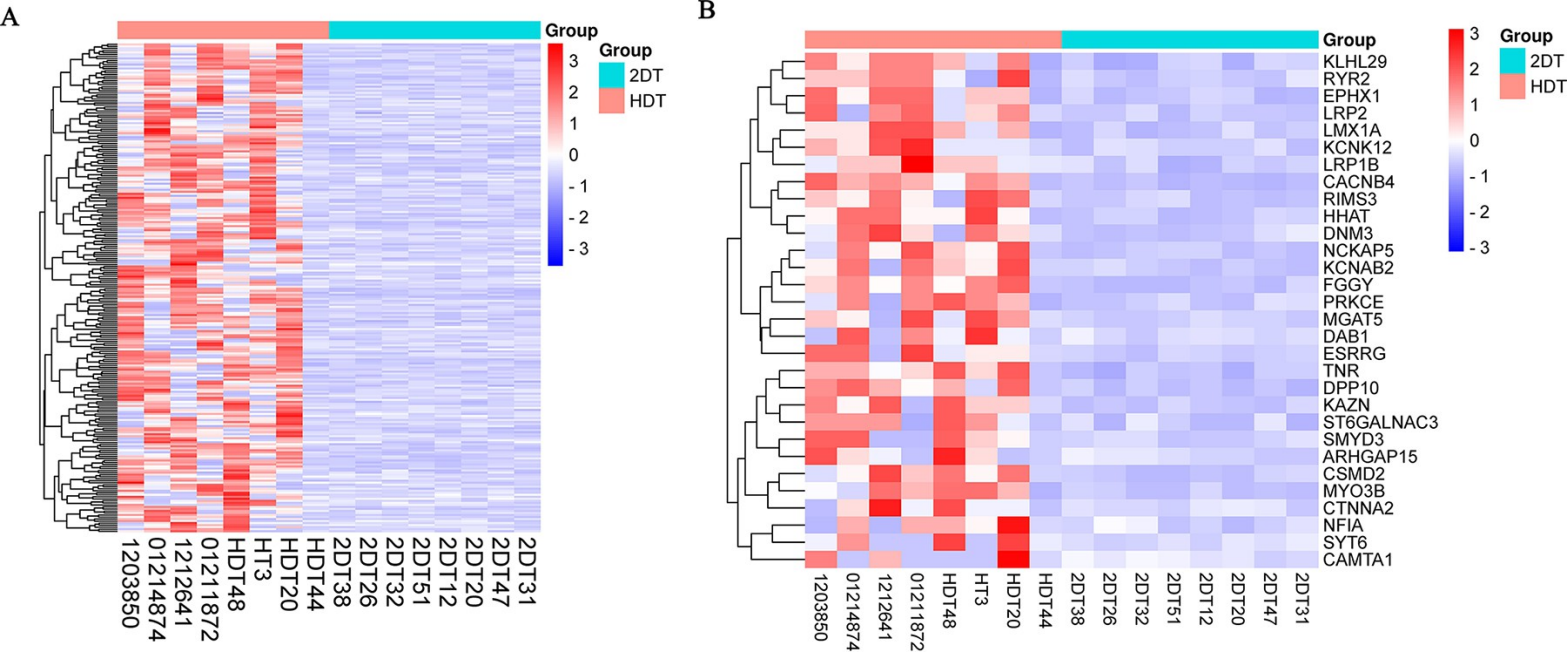
(A) Cluster analysis of 243 differential SNP genes. (B) Cluster analysis of top 30 differential SNP genes.

SNP microarray analysis revealed 14596 SNP loci in patients with DR relative to the control group. Nine genes were associated with DR, upon comparison of the DR-associated predicted genes with the GeneCards database. S2 Table lists the CHB frequencies in the HapMap database. The SNP mutation rate was significantly higher in DR patients than in controls (Fig 1A and 1B).

### Gene Ontology (GO) and pathway analyses

**Fig 2:**
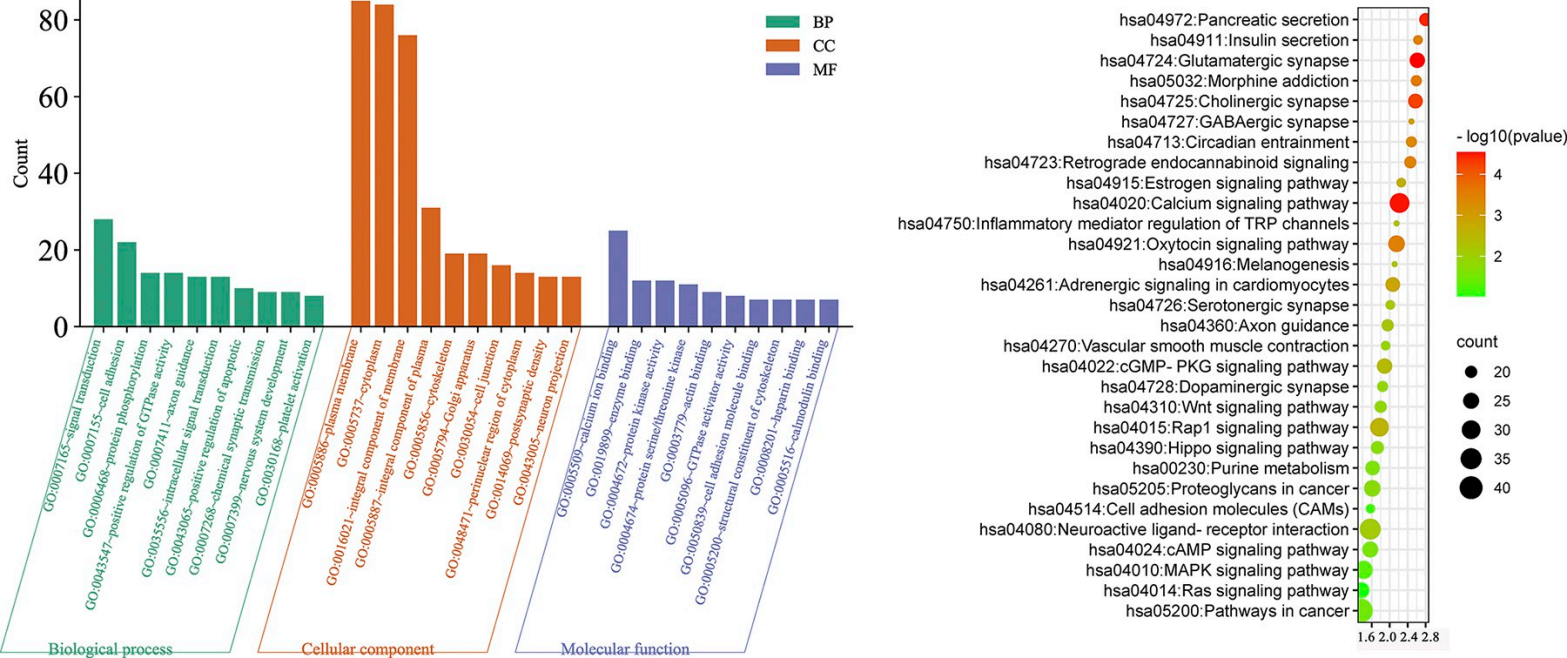
Functional categories of SNPs. (A) Gene ontology analysis showed that the mutated SNPS were mainly involved in biological processes such as signal transduction, cell adhesion, protein phosphorylation, positive regulation of GTPase activity, axon guidance, intracellular signal transduction, positive regulation of apoptotic, chemical synaptic transmission, nervous system development, and platelet activation. (B) KEGG pathway analysis showed that the mutated SNPs were mainly enriched in PI3K/AKT, calcium pathway, and glutamatergic synapse and cholinergic synapse signaling pathways.

To explore the functional categories of SNPs, we performed GO and Kyoto Encyclopedia of Genes and Genomes (KEGG) analyses using the KOBAS 2.0 server. Hypergeometric test and Benjamini-Hochberg false discovery rate (FDR) controlling procedure were applied to define the enrichment of each term. The mutated SNPs were mainly enriched in PI3K/AKT, calcium pathway, and glutamatergic synapse and cholinergic synapse signaling pathways (Fig 2A and 2B).

### Validation of the differences in SNPs

We clarified the differences in the SNPs between the case and control groups in a larger sample size to validate the involvement of these eight SNPs, including PRKCE.rs1533476, DNAH11.rs10485983, ERAP1.rs149481, KLHL1.rs1318761, APOBEC3C.rs1969643, FYN.rs11963612, KCTD1.rs7240205, and PIK3CA.rs17849079. We eliminated CDH4 because it failed to query the allele frequency in the NCBI database.

### Association of SNPs with DR

According to the Hardy-Weinberg genetic equilibrium testing, the frequencies of the eight SNPs were substituted to obtain genotype equilibrium frequencies (Table 1). The genotype distributions of rs17849079 and rs1969643 loci were significantly different between the case and control group (p<0.05) (Table 2). The T allele of PIK3CA.rs17849079 was significantly higher than others, and may act as a protective factor in DR (Table 3).

**Table 1:** Eight SNPs subjected to Hardy-Weinberg equilibrium.

SNP	Allele	Group	Frequency of genotype observed			HWp
	(1/2)		1/1	1/2	2/2	
rs149481	T/G	Case (n = 56)	23 (25.79)	30 (24.43)	3 (5.79)	0.088
		Control (n = 58)	27 (26.9)	25 (25.2)	6 (5.9)	0.952
rs1318761	G/A	Case (n = 56)	23 (22.5)	25 (25.99)	8 (7.5)	0.775
		Control (n = 58)	16 (17.11)	31 (28.78)	11 (12.11)	0.558
rs1969643	T/C	Case (n = 55)	9 (13.5)	35 (27.99)	11 (14.5)	0.061
		Control (n = 55)	21 (19.93)	26 (28.14)	8 (9.93)	0.563
rs10485983	T/C	Case (n = 56)	23 (24.45)	28 (25.11)	5 (6.45)	0.389
		Control (n = 58)	26 (27.59)	28 (24.83)	4 (5.59)	0.330
rs11963612	C/T	Case (n = 56)	25 (27.86)	29 (23.28)	2 (4.86)	0.066
		Control (n = 58)	29 (28.98)	24 (24.03)	5 (4.98)	0.991
rs7240205	C/T	Case (n = 56)	17 (16.61)	27 (27.78)	12 (11.61)	0.834
		Control (n = 58)	23 (26.92)	23 (27.96)	12 (9.52)	0.177
rs1533476	T/C	Case (n = 52)	28 (19.61)	8 (7.69)	14 (13.46)	0.186
		Control (n = 55)	34 (30.91)	4 (3.64)	19 (17.27)	0.069
rs17849079	T/C	Case (n = 157)	87 (0.55)	54 (0.34)	16 (0.10)	0.11
		Control (n = 96)	67 (0.70)	28 (0.29)	1 (0.01)	0.45

**Table 2:** Genotype distribution of SNPs in case and control groups.

SNP	Allele	Group	Genotype, n (%)			χ^2^	*P*
	(1/2)		1/1	½	2/2		
rs149481	T/G	Case (n = 56)	23 (0.411)	30 (0.536)	3 (0.054)	1.740	0.419
		Control (n = 58)	27 (0.466)	25 (0.431)	6 (0.103)		
rs1318761	G/A	Case (n = 56)	23 (0.411)	25 (0.446)	8 (0.143)	2.339	0.311
		Control (n = 58)	16 (0.276)	31 (0.534)	11 (0.190)		
rs1969643	T/C	Case (n = 55)	9 (0.164)	35 (0.636)	11 (0.200)	6.602	0.037
		Control (n = 55)	21 (0.382)	26 (0.473)	8 (0.145)		
rs10485983	T/C	Case (n = 56)	23 (0.411)	28 (0.500)	5 (0.089)	0.260	0.878
		Control (n = 58)	26 (0.448)	28 (0.483)	4 (0.069)		
rs11963612	C/T	Case (n = 56)	25 (0.446)	29 (0.518)	2 (0.036)	2.019	0.364
		Control (n = 58)	29 (0.500)	24 (0.414)	5 (0.086)		
rs7240205	C/T	Case (n = 56)	17 (0.304)	27 (0.482)	12 (0.214)	1.185	0.553
		Control (n = 58)	23 (0.397)	23 (0.397)	12 (0.207)		
rs1533476	T/C	Case (n = 52)	29 (0.551)	9 (0.163)	14 (0.286)	2.304	0.316
		Control (n = 55)	36 (0.596)	4 (0.070)	19 (0.333)		
rs17849079	T/C	Case (n = 157)	87 (0.55)	54 (0.34)	16 (0.10)	9.947	0.006
		Control (n = 96)	67 (0.70)	28 (0.29)	1 (0.01)		

**Table 3:** Genotype and allele frequencies of the eight SNPs in case and control groups.

SNP	Allele	Case	Control	χ^2^	*P*	OR	%95CI
rs149481	G	36 (0.321)	37 (0.319)	0.002	0.968	1.011	[0.574~1.764]
	T	76 (0.679)	79 (0.681)			1	ref
rs1318761	A	41 (0.366)	53 (0.457)	1.940	0.164	0.686	[0.401~1.166]
	G	71 (0.634)	63 (0.543)			1	ref
rs1969643	C	57 (0.518)	42 (0.382)	2.076	0.150	1.468	[0.878~2.477]
	T	53 (0.482)	68 (0.618)			1	ref
rs10485983	C	38 (0.339)	36 (0.31)	0.218	0.641	1.141	[0.656~1.987]
	T	74 (0.661)	80 (0.69)			1	ref
rs11963612	C	79 (0.705)	82 (0.707)	0.001	0.980	0.993	[0.567~1.755]
	T	33 (0.295)	34 (0.293)			1	ref
rs7240205	C	61 (0.545)	69 (0.595)	0.586	0.444	0.815	[0.481~1.377]
	T	51 (0.455)	47 (0.405)			1	ref
rs1533476	C	36 (0.367)	42 (0.368)	0.000	0.987	0.995	[0.560~1.742]
	T	62 (0.633)	72 (0.632)			1	ref
rs17849079	C	86 (0.27)	30 (0.16)	9.947	0.0022	0.51	[0.32~0.81]
	T	228 (0.73)	162 (0.84)				

SNPs related to the prognosis of DR. Univariate Cox regression analysis forest plot. Multivariate Cox regression analysis forest plot. In comparison with reference samples, samples with a hazard ratio greater than 1 had a higher risk of DR and those with a hazard ratio less than 1 had a lower risk of DR.

### Results of cell experiments

According to the results of sequencing and validation, PIK3CA.rs17849079 was found to be associated with the development of DR. Therefore, we established a DR cell model by treating ARPE-19 cells with high glucose, followed by their transfection with a plasmid carrying wild-type and mutated PIK3CA.rs17849079, to explore the relationship between PIK3CA.rs17849079 and DR development.

### Flow cytometry and cytokine analysis

**Fig 3:**
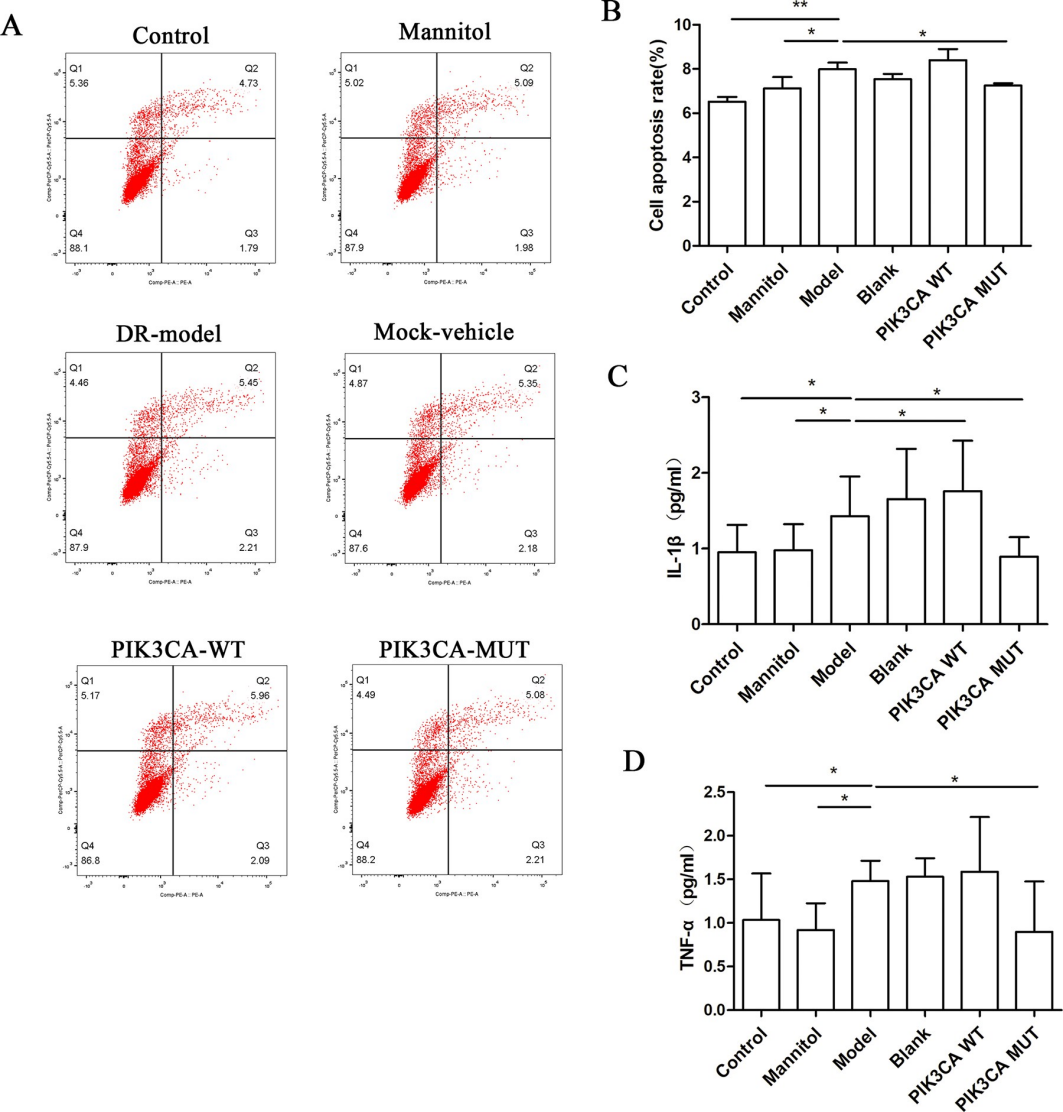
PIK3CA and glucose treatment activated apoptosis and induced secretion of soluble cytokines. A, Flow cytometry profiles of each group of transfected cells. B, The most severe apoptosis was observed in the PIK3CA-wt group, followed by the DR model group. The PIK3CA-mut group had lower apoptosis than the wild-type group. C, The secretion of IL-1β was the highest in the PIK3CA.rs17849079-wt group, followed by the DR model group. D, The secretion of TNF-α was the highest in the PIK3CA.rs17849079-wt group, followed by the DR model group.(*: p<0.05, **: p<0.01).

We detected the trends in cellular apoptosis in each group by flow cytometry, and determined the levels of TNF-α and IL-1β by ELISA kits. The results are shown in Fig 3.

### Expression of mRNAs was detected by RT-qPCR

**Fig 4:**
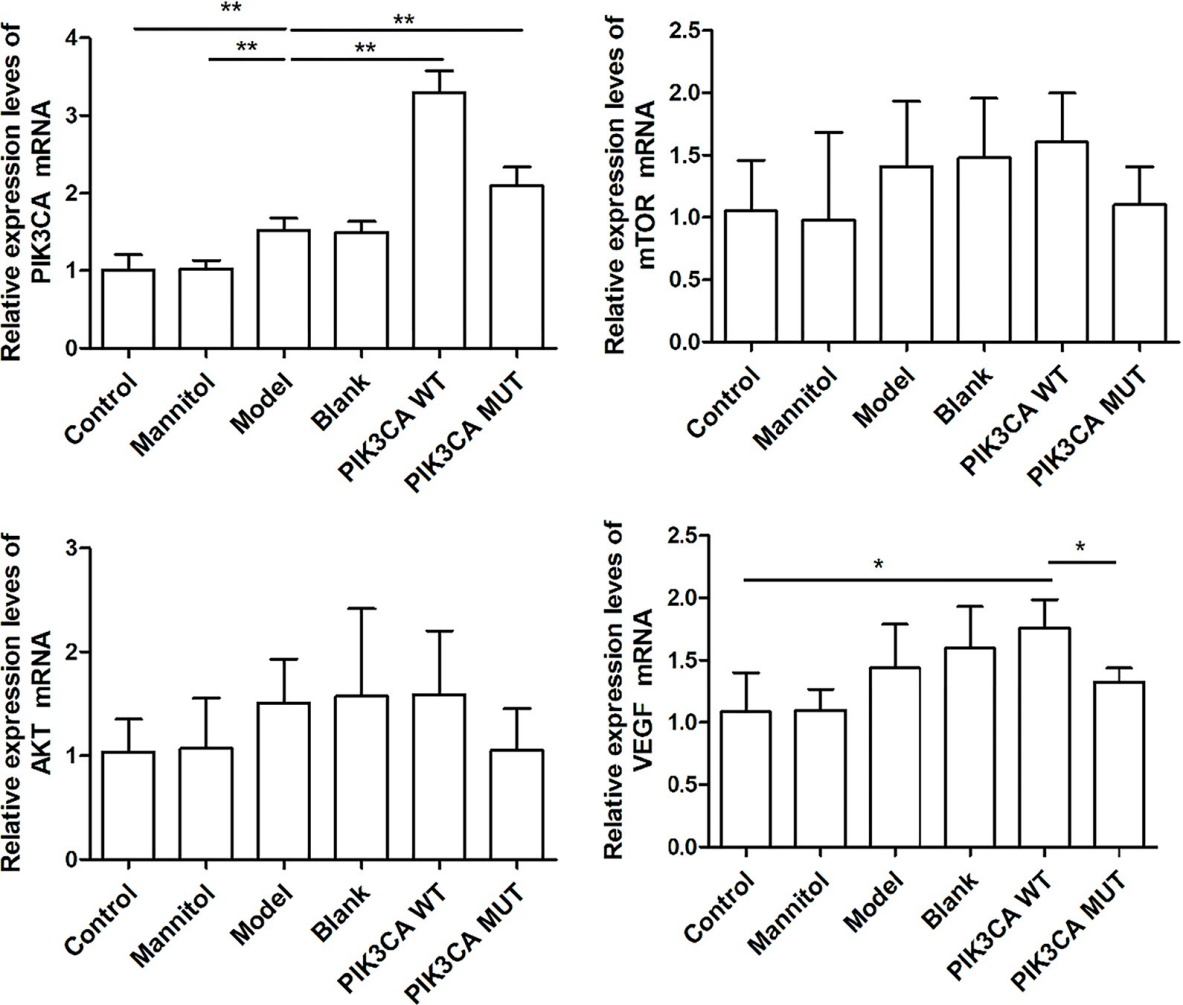
The mRNA expression in each group. A, *PIK3CA* mRNA expression was the highest in the PIK3CA-wt group, followed by PIK3CA-mut and DR model groups. B, *mTOR* mRNA expression was not significantly different between groups. C, *AKT* mRNA expression was not significantly different between the groups. D, *VEGF* mRNA expression was the highest in the PIK3CA-wt group, followed by the empty vector group, PIK3CA-mut group, and DR model group (*, p<0.05).

We performed RT-qPCR analysis to detect expression trends in *PIK3CA*, *mTOR*, *AKT*, and *VEGF* mRNAs in each group; the results are shown in Fig 4.

### Expression of proteins was detected by western blotting

**Fig 5:**
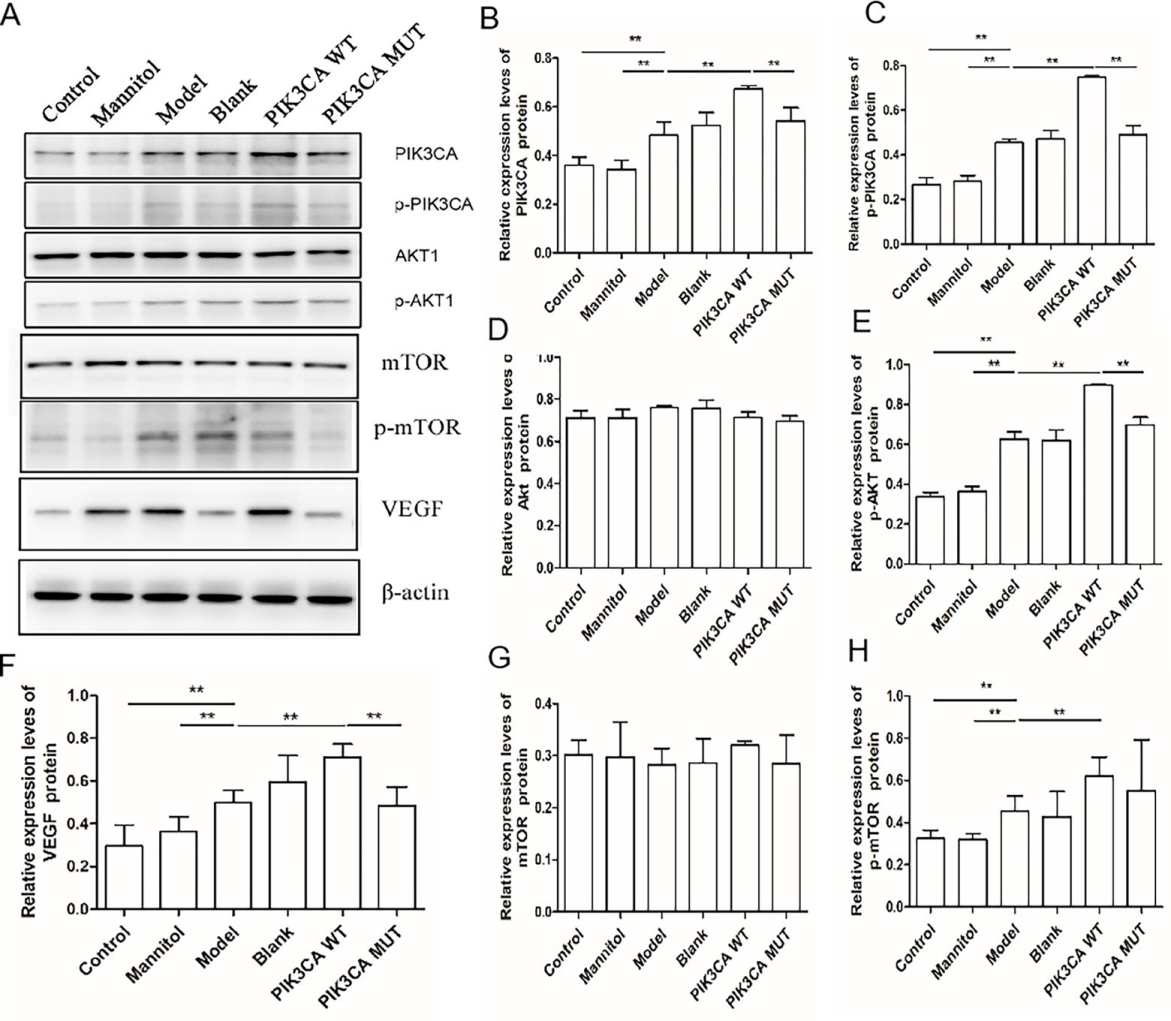
Protein expression. A, Protein expression in each group. B, PI3K expression was the highest in the PIK3CA-wt group, followed by PIK3CA-mut and DR model groups. B, mTOR protein expression was not significantly different between the groups. C, No significant difference was observed in AKT protein expression between different groups. D, VEGF protein expression was the highest in the PIK3CA-wt group, followed by PIK3CA-mut and DR model groups (*, p<0.05).

The trends in the expression of PI3K, p-PI3K, AKT, p-AKT, mTOR, p-mTOR, and VEGF proteins in each group were detected by western blotting, and the results are shown in Fig 5.

## Discussion

DR is a complex disease involving multiple biological processes [8]. The retina of DR patients is in high glucose state, which stimulates the production of various cytokines, including VEGF, TGF-β, and bone morphogenic proteins (BMPs). These cytokines degrade the vascular basement membrane and promote the proliferation and migration of endothelial cells [9]. In this study, we found a susceptible C\T mutation at rs17849079 in *PIK3CA* by sequencing and validation. The risk of DR increased with the presence of the C allele, while the T allele had the opposite effect. By comparing with SNP library in the National Centre for Biotechnology Information (NCBI) database, we found that the PIK3CA.rs17849079 T allele was a protective factor. PRKCE.rs1533476, DNAH11.rs10485983, ERAP1.rs149481, KLHL1.rs1318761, APOBEC3C.rs1969643, FYN. rs11963612, and KCTD1.rs7240205 were not associated with the development of DR. GO and KEEG enrichment analyses revealed the involvement of the mutated SNPs in PI3K/AKT, calcium ion, and glutamatergic synapse and cholinergic synapse signal transduction pathways. Therefore, we used high glucose-induced ARPE-19 cells as a DR model carrying a PIK3CA rs17849079 C/T site-directed mutation to explore the underlying mechanism.

The *PIK3CA* gene encodes PI3K, a 124 kDa protein comprising 1068 amino acid residues. It is a heterodimeric enzyme consisting of a p110α catalytic subunit and a p85 regulatory subunit [10, 11]. Upon activation by receptor tyrosine kinases (RTKs), PI3K can phosphorylate phosphatidylinositol 4,5-bisphosphate (PIP2) to the second messenger phosphatidylinositol (3,4,5)-trisphosphate (PIP3) [12, 13] and initiate multiple downstream pathways. These pathways are mainly mediated by AKT, mTOR, and other downstream factors [14, 15] and play an important role in many processes, including cell proliferation, angiogenesis, cell survival, and metabolism [16, 17].

AKT belongs to the serine/threonine protein kinase family [18], which can activate PI3K and participate in biological processes such as cell survival, proliferation, DNA repair, and anabolism [19–21] by regulating the phosphorylation of different substrates [22]. mTOR, a serine/threonine protein kinase, is the central hub of cell metabolism, and is involved in amino acid, glucose, nucleotide, fatty acid, and lipid metabolism as well as other biological processes. Glucose activates mTOR, which in turn promotes glucose uptake by AKT phosphorylation [23, 24].

We used ARPE-19 cells to establish a DR cell model, which was employed to investigate high and low expression of the *PIK3CA* gene rs17849079 locus. High glucose promoted ARPE-19 cell apoptosis, inhibited cell proliferation, and increased glucose uptake, which is consistent with previous results where high glucose promoted cell apoptosis [25, 26], inhibited cell mitosis and proliferation, and had a dose-dependent effect on retinal pigment epithelium (RPE) [27, 28].

Il-1β is an important member of the IL-1 family that exerts strong pro-inflammatory activity and induce the production of a variety of pro-inflammatory mediators [29]. TNF-α is a cytokine with pleiotropic effects on different cell types. It is the main regulator of an inflammatory response and is involved in the pathogenesis of some inflammatory and autoimmune diseases. This study found that the levels of TNF-α and IL-1β increased in the ARPE-19 DR cell model, possibly because inflammation is one of the fundamental factors in various pathophysiological processes and involved in all stages of DR development. In this study, the mRNA expression of *PIK3CA* and *VEGF* was upregulated in the model group, but that of *AKT1* and *mTOR* was not significantly different among the groups. Thus, high glucose could induce the mRNA expression of *PIK3CA* and *VEGF* genes. In comparison with the DR model group, the PIK3CA-wt group showed increased apoptosis, higher suppression of cell proliferation, enhanced glucose uptake, and upregulated TNF-α and IL-1β levels. Further, the expression of *VEGF* showed an upward trend. This observation is consistent with previous findings where overexpression of *PIK3CA* accelerated glucose transport [30] and played a critical role in cell metabolism and growth [31]. In comparison with the PIK3CA-wt group, the PIK3CA-mut group showed decreased glucose update, lower levels of TNF-α and IL-1β, reduced cellular apoptosis, and higher proliferation; their expression of *VEGF* showed a downward trend. In conclusion, the C allele of PIK3CA.rs17849079 may promote the expression of *VEGF*, reduce the proliferation ability of cells, accelerate their apoptosis, and promote the uptake of glucose and secretion of inflammatory factors TNF-α and IL-1β. If the C allele was mutated to the T allele, the expression of *VEGF* was inhibited and the proliferation ability of ARPE-19 cells increased, consistent with a decrease in the level of apoptosis, glucose uptake, and inflammatory cytokines TNF-α and IL-1β. To further investigate the underlying mechanism, we performed western blot analysis.

Western blotting results revealed the upregulation in the expression of PI3K, p-PI3K, p-AKT1, p-mTOR, and VEGF in the DR model group, which indicates that high glucose promoted the phosphorylation of PI3K, AKT1, and mTOR and consequently increased the expression of VEGF. In the PIK3CA-wt group, PI3K, p-PI3K, p-AKT1, p-mTOR, and VEGF expression showed an upward trend, indicating that *PIK3CA* promoted the phosphorylation of PI3K, AKT1, and mTOR and eventually increased VEGF expression. When the C allele of PIK3CA.rs17849079 was mutated to T, p-PI3K, p-AKT1, p-mTOR, and VEGF expression showed a downward trend and VEGF expression significantly decreased. This result indicates that PIK3CA.rs17849079 mutation may inhibit the phosphorylation of PI3K, AKT1, and mTOR and eventually decrease the expression of VEGF.

In conclusion, high glucose induces the expression of PIK3CA.rs17849079, which may lead to the phosphorylation of PI3K, AKT, and mTOR and an eventual increase in VEGF expression and development of DR. If the C allele of *PIK3CA* gene rs17849079 was mutated to T allele, these biological processes were inhibited and ARPE-19 cells were protected.

This study has some limitations such as the low sample size of clinical samples. Hence, it is necessary to further expand the sample size in future studies. While the human retinal pigment epithelial cell DR model was established for site-directed mutagenesis, only in vitro experiments were carried out and in vivo experiments are warranted to further verify the relationship between PIK3CA.rs17849079 and the occurrence and development of DR. These limitations will be addressed in the follow-up research.

Thus, early screening of risk-associated genes for DR can plausibly predict subclinical DR and prevent the development of N-STDR to STDR. This strategy may serve as a new mode to prevent the occurrence and development of DR.

## Supporting information

S1 Table Primer sequence. (DOCX)

S2 Table CHB frequencies in the Hapmap database. (DOCX)

## References

[1] Al-Kharashi AS. Role of oxidative stress, inflammation, hypoxia and angiogenesis in the development of diabetic retinopathy. Saudi J Ophthalmol. 2018;32(4):318–323. doi: 10.1016/j.sjopt.2018.05.002 30581303 PMC6300752

[2] Behl T, Kaur I, Kotwani A. Role of endocannabinoids in the progression of diabetic retinopathy. Diabetes Metab Res Rev. 2016;32(3):251–259. doi: 10.1002/dmrr.2710 26379208

[3] Sorrentino FS, Matteini S, Bonifazzi C, et al. Diabetic retinopathy and endothelin system: microangiopathy versus endothelial dysfunction. Eye (Lond). 2018;32(7):1157–1163. doi: 10.1038/s41433-018-0032-4 29520046 PMC6043602

[4] Kern TS, Antonetti DA, Smith LEH. Pathophysiology of Diabetic Retinopathy: Contribution and Limitations of Laboratory Research. Ophthalmic Res. 2019;62(4):196–202. doi: 10.1159/000500026 31362288 PMC6872907

[5] Joussen AM, Poulaki V, Qin W, et al. Retinal vascular endothelial growth factor induces intercellular adhesion molecule-1 and endothelial nitric oxide synthase expression and initiates early diabetic retinal leukocyte adhesion in vivo. Am J Pathol. 2002;160(2):501–509. doi: 10.1016/S0002-9440(10)64869-9 11839570 PMC1850650

[6] Wells JA, Glassman AR, Ayala AR, et al. Aflibercept, Bevacizumab, or Ranibizumab for Diabetic Macular Edema: Two-Year Results from a Comparative Effectiveness Randomized Clinical Trial. Ophthalmology. 2016;123(6):1351–1359. doi: 10.1016/j.ophtha.2016.02.022 26935357 PMC4877252

[7] Zhao Kanxing, Yang Peizeng. Ophthalmology [M]. People’s Medical Publishing House. 2008:197.

[8] Esfahanian N, Shakiba Y, Nikbin B, et al. Effect of metformin on the proliferation, migration, and MMP-2 and -9 expression of human umbilical vein endothelial cells. Mol Med Rep. 2012;5(4):1068–1074. doi: 10.3892/mmr.2012.753 22246099 PMC3493092

[9] Lutty GA, McLeod DS, Merges C, et al. Localization of vascular endothelial growth factor in human retina and choroid. Arch Ophthalmol. 1996;114(8):971–977. doi: 10.1001/archopht.1996.01100140179011 8694733

[10] Kobialka P, Graupera M. Revisiting PI3-kinase signalling in angiogenesis. Vasc Biol. 2019;1(1):H125–H134. Published 2019 Nov 29. doi: 10.1530/VB-19-0025 32923964 PMC7439845

[11] Alqahtani A, Ayesh HSK, Halawani H. PIK3CA Gene Mutations in Solid Malignancies: Association with Clinicopathological Parameters and Prognosis. Cancers (Basel). 2019;12(1):93. Published 2019 Dec 30. doi: 10.3390/cancers12010093 31905960 PMC7017171

[12] Bilanges B, Posor Y, Vanhaesebroeck B. PI3K isoforms in cell signalling and vesicle trafficking. Nat Rev Mol Cell Biol. 2019;20(9):515–534. doi: 10.1038/s41580-019-0129-z 31110302

[13] Parker VER, Keppler-Noreuil KM, Faivre L, et al. Safety and efficacy of low-dose sirolimus in the PIK3CA-related overgrowth spectrum. Genet Med. 2019;21(5):1189–1198. doi: 10.1038/s41436-018-0297-9 30270358 PMC6752269

[14] Wassef M, Blei F, Adams D, et al. Vascular Anomalies Classification: Recommendations From the International Society for the Study of Vascular Anomalies. Pediatrics. 2015;136(1):e203–e214. doi: 10.1542/peds.2014-3673 26055853

[15] Keppler-Noreuil KM, Parker VE, Darling TN, et al. Somatic overgrowth disorders of the PI3K/AKT/mTOR pathway & therapeutic strategies. Am J Med Genet C Semin Med Genet. 2016;172(4):402–421. doi: 10.1002/ajmg.c.31531 27860216 PMC5592089

[16] Nguyen HL, Boon LM, Vikkula M. Vascular Anomalies Caused by Abnormal Signaling within Endothelial Cells: Targets for Novel Therapies. Semin Intervent Radiol. 2017;34(3):233–238. doi: 10.1055/s-0037-1604296 28955112 PMC5615384

[17] Leroux AE, Schulze JO, Biondi RM. AGC kinases, mechanisms of regulation ‎and innovative drug development. Semin Cancer Biol. 2018;48:1–17. doi: 10.1016/j.semcancer.2017.05.011 28591657

[18] Humphrey SJ, Yang G, Yang P, et al. Dynamic adipocyte phosphoproteome reveals that Akt directly regulates mTORC2. Cell Metab. 2013;17(6):1009–1020. doi: 10.1016/j.cmet.2013.04.010 23684622 PMC3690479

[19] Humphrey SJ, Azimifar SB, Mann M. High-throughput phosphoproteomics reveals in vivo insulin signaling dynamics. Nat Biotechnol. 2015;33(9):990–995. doi: 10.1038/nbt.3327 26280412

[20] Manning BD, Toker A. AKT/PKB Signaling: Navigating the Network. Cell. 2017;169(3):381–405. doi: 10.1016/j.cell.2017.04.001 28431241 PMC5546324

[21] Toker A, Marmiroli S. Signaling specificity in the Akt pathway in biology and disease. Adv Biol Regul. 2014;55:28–38. doi: 10.1016/j.jbior.2014.04.001 24794538 PMC4062840

[22] Risso G, Blaustein M, Pozzi B, et al. Akt/PKB: one kinase, many modifications. Biochem J. 2015;468(2):203–214. doi: 10.1042/BJ20150041 25997832

[23] Mossmann D, Park S, Hall MN. mTOR signalling and cellular metabolism are mutual determinants in cancer. Nat Rev Cancer. 2018;18(12):744–757. doi: 10.1038/s41568-018-0074-8 30425336

[24] Yuan T, Lupse B, Maedler K, et al. mTORC2 Signaling: A Path for Pancreatic β Cell’s Growth and Function. J Mol Biol. 2018;430(7):904–918. doi: 10.1016/j.jmb.2018.02.013 29481838

[25] Maugeri G, D’Amico AG, Bucolo C, et al. Protective effect of PACAP-38 on retinal pigmented epithelium in an in vitro and in vivo model of diabetic retinopathy through EGFR-dependent mechanism. Peptides. 2019;119:170108. doi: 10.1016/j.peptides.2019.170108 31247223

[26] Zha X, Xi X, Fan X, et al. Overexpression of METTL3 attenuates high-glucose induced RPE cell pyroptosis by regulating miR-25-3p/PTEN/Akt signaling cascade through DGCR8. Aging (Albany NY). 2020;12(9):8137–8150. doi: 10.18632/aging.103130 32365051 PMC7244028

[27] Dinarello CA. Interleukin-1 in the pathogenesis and treatment of inflammatory diseases. Blood. 2011;117(14):3720–3732. doi: 10.1182/blood-2010-07-273417 21304099 PMC3083294

[28] Scuderi S, D’Amico AG, Castorina A, et al. Ameliorative effect of PACAP and VIP against increased permeability in a model of outer blood retinal barrier dysfunction. Peptides. 2013;39:119–124. doi: 10.1016/j.peptides.2012.11.015 23220033

[29] Jang DI, Lee AH, Shin HY, et al. The Role of Tumor Necrosis Factor Alpha (TNF-α) in Autoimmune Disease and Current TNF-α Inhibitors in Therapeutics. Int J Mol Sci. 2021;22(5):2719. doi: 10.3390/ijms22052719 33800290 PMC7962638

[30] Mitchell CB, Phillips WA. Mouse Models for Exploring the Biological Consequences and Clinical Significance of PIK3CA Mutations. Biomolecules. 2019;9(4):158. doi: 10.3390/biom9040158 31018529 PMC6523081

[31] Luks VL, Kamitaki N, Vivero MP, et al. Lymphatic and other vascular malformative/overgrowth disorders are caused by somatic mutations in PIK3CA. J Pediatr. 2015;166(4):1048–54.e545. doi: 10.1016/j.jpeds.2014.12.069 25681199 PMC4498659

