# Supplementary material for: PIK3CA regulates development of diabetes retinopathy through the PI3K/Akt/mTOR pathway
Source: PLoS One. 2024 Jan 9;19(1):e0295813. doi: 10.1371/journal.pone.0295813 (PMC10775978; doi:10.1371/journal.pone.0295813)
Supplement: S1 Table — (DOCX) [file pone.0295813.s001.docx]

Table S1  [Primer sequence](javascript:;)

| Primer | Sequence | Fragment  Size | Annealing Temperature | Extension Time |
| --- | --- | --- | --- | --- |
| rs149481-F1 | AATAGTGTCCTAACTGGCCTCCC | 368bp | 56.7℃ | 24s |
| rs149481-R1 | AAGATAAAATGCCCCTTTCTAGTGA |  |  |  |
| rs1318761-F2 | CCCAGCCTTATTTTTAGTTTCCA | 352bp | 56.7℃ | 24s |
| rs1318761-R2 | GCATCCAGCGTCTTTCCATAG |  |  |  |
| rs1969643-F1 | AAAATTAGGCAGGTGTGGCGG | 291bp | 59.5℃ | 24s |
| rs1969643-R1 | TCTCAGAATCCACCTGGCGAAG |  |  |  |
| rs10485983-F1 | TTATCTTAGAAGGGCGTGTTGGT | 471bp | 59.5℃ | 30s |
| rs10485983-R1 | AGGCTTCATCAGAGGCATTTTG |  |  |  |
| rs11963612-F1 | CCACATCCGGCTAACTTTTGCAT | 436bp | 56.7℃ | 30s |
| rs11963612-R1 | CCAAGTATTTGCCATCTGCTTTCAC |  |  |  |
| rs7240205-F2 | CTCCCTAAAGCCTCATTGAAAGC | 477bp | 56.7℃ | 30s |
| rs7240205-R2 | GTACTAATCACAGGAGAAACAGCGA |  |  |  |
| rs1533476-F3 | TTGAGACTGACCCAGGTTGTTT | 464bp | 56.7℃ | 30s |
| rs1533476-R3 | TATAGGCCTTGTTCATCTCCTCATT |  |  |  |
| rs17849079-F3 | GTTTCAGGAGATGTGTTACA | 458bp | 56.7℃ | 30s |
| rs17849079-R3 | CAGCCACCATGATGTGCATCA |  |  |  |
