# Supplementary material for: PIK3CA regulates development of diabetes retinopathy through the PI3K/Akt/mTOR pathway
Source: PLoS One. 2024 Jan 9;19(1):e0295813. doi: 10.1371/journal.pone.0295813 (PMC10775978; doi:10.1371/journal.pone.0295813)
Supplement: S2 Table — (DOCX) [file pone.0295813.s002.DOCX]

Table S2 CHB frequencies in the Hapmap database

|  | SNP | Allele | [Genotype Frequency](javascript:;) | | | [Allele Frequency](javascript:;) | |
| --- | --- | --- | --- | --- | --- | --- | --- |
|  |  | 1/2 | 1/2 | 2/2 | 1/1 | 1 | 2 |
| PRKCE | rs1533476 | [T/C] | 0.488 | 0.244 | 0.268 | 0.512 | 0.488 |
| CDH4 | rs58380524 | [T/C] |  |  |  |  |  |
| DNAH11 | rs10485983 | [T/C] | 0.463 | 0.122 | 0.415 | 0.646 | 0.354 |
| ERAP1 | rs149481 | [A/C] | 0.366 | 0.024 | 0.61 | 0.793 | 0.207 |
| KLHL1 | rs1318761 | [T/C] | 0.415 | 0.097 | 0.188 | 0.695 | 0.305 |
| APOBEC3C | rs1969643 | [T/C] | 0.592 | 0.122 | 0.306 | 0.602 | 0.398 |
| FYN | rs11963612 | [T/C] | 0.238 | 0.4131 | 0.3489 | 0.3489 | 0.6511 |
| KCTD1 | rs7240205 | [C/T] | 0.327 | 0.2474 | 0.4256 | 0.5744 | 0.4726 |
| PIK3CA | rs17849079 | [C/T] | 0.008 | 0.001 | 0.991 | 0.995 | 0.005 |
